# Reduction of hepatic fibrosis by overexpression of von Hippel–Lindau protein in experimental models of chronic liver disease

**DOI:** 10.1038/srep41038

**Published:** 2017-01-23

**Authors:** Jizhou Wang, Zhaoyang Lu, Zhilin Xu, Pei Tian, Hui Miao, Shangha Pan, Ruipeng Song, Xueying Sun, Baolei Zhao, Dawei Wang, Yong Ma, Xuan Song, Shugeng Zhang, Lianxin Liu, Hongchi Jiang

**Affiliations:** 1 Key Laboratory of Hepatosplenic Surgery, Department of Hepatic Surgery, The First Affiliated Hospital of Harbin Medical University, Harbin 150001, China; 2 Department of Pediatric Surgery, The First Affiliated Hospital of Harbin Medical University, Harbin 150001, China; 3 Department of Ophthalmology, The First Affiliated Hospital of Harbin Medical University, Harbin 150001, China; 4 Department of Molecular Medicine and Pathology, School of Medical Sciences, University of Auckland, Auckland 1023, New Zealand

## Abstract

Hypoxia-inducible factor (HIF)-1α and HIF-2α play an important role in liver fibrosis. von Hippel–Lindau protein (VHL), a key mediator of HIF-α, regulates fibrosis in an organ- and cell-specific way. In this study, human liver samples were collected from hepatitis C-, alcoholic-, and cholestatic-associated fibrotic and healthy individuals. Two mouse models of liver fibrosis were established: bile duct ligation and carbon tetrachloride injection. We constructed adenovirus vectors to overexpress VHL, normoxia-active HIF-α, and lentiviral vectors to silence HIF-α. The results showed that liver sections from fibrosis patients had a lower level of VHL and higher levels of HIF-1α and HIF-2α compared with healthy sections, a finding which was confirmed in mice. Overexpression of VHL attenuated liver fibrosis, downregulated fibrogenic genes, and inhibited liver inflammation, apoptosis, and angiogenesis. Overexpression of VHL was more successful at inhibiting fibrosis compared with silencing HIF-1α plus HIF-2α. Normoxia-active HIF-1α or HIF-2α prevented the inhibitory effect of VHL on liver fibrosis, indicating that attenuating fibrosis via VHL is HIF-1α- and HIF-2α-dependent to some extent. In addition, overexpression of VHL inhibited mouse hepatic stellate cells activation and proliferation and promoted apoptosis. Taken together, VHL may be considered a new target to inhibit liver fibrosis.

Despite the liver’s capacity to regenerate, chronic or overwhelming injury often causes liver fibrosis, which can culminate in cirrhosis and hepatic failure^1^. Unfortunately, we still lack effective antifibrotic therapies^2^. Hepatic stellate cells (HSCs), a pericyte-like cell population in the liver, are widely considered the most relevant source of hepatic myofibroblasts^3^.

Hypoxia has a role in the pathogenesis of several forms of liver disease, including ischemia-reperfusion injury, hepatocellular carcinoma (HCC), and particularly liver fibrosis^4^. Hypoxia-inducible factors (HIFs) are a family of evolutionarily conserved transcriptional regulators that have a homeostatic response to low oxygen tension. HIFs consist of an oxygen-dependent α subunit (HIF-1α, HIF-2α, or HIF-3α), a constitutively expressed β subunit and aryl hydrocarbon nuclear translocator (ARNT). Inactivation of von Hippel-Lindau (VHL) gene predisposes patients to several organ-specific benign and malignant tumors, including hemangioblastoma and clear-cell renal cell carcinoma. The gene product of VHL, which is a multifunctional adaptor protein, is the substrate-recognition subunit in an E3 ubiquitin ligase^5^. In cells, adequate oxygen levels cause prolyl hydroxylation of HIF-α subunits, an activity that is required for HIF-α to bind to VHL, leading to ubiquitination and degradation of HIF-α^6^. A decrease in cellular oxygen or inactivation of VHL results in stabilization of HIF-α to activate the transcription of genes that regulate the response to hypoxia. HIF-1α and HIF-2α regulate distinct but overlapping target genes^7^. HIF-1α and HIF-2α play an important role in fibrosis which can be either beneficial or deleterious depending on the timing and situation. Stable expression of HIF-1α in tubular epithelial cells promotes renal interstitial fibrosis^8^. Another study showed that sustained overexpression of HIF-2α alone is sufficient to induce tubulointerstitial fibrosis and renal insufficiency^9^. Recent evidence indicates that HIF-1α is activated in the liver subjected to bile duct ligation (BDL), whereas liver fibrosis is reduced in HIF-1α-deficient mice^10^. Later, the same group reported that profibrotic mediators were induced by hypoxic hepatocytes, which only partially prevented in HIF-1α-null cells, suggesting that other HIF isoforms (particularly HIF-2α) may play a role^11^. More recently, another group reported that HIF-2α promotes liver steatohepatitis through augmenting lipid accumulation and inflammation^12^.

VHL, a key regulator of HIF-α, also plays a role in fibrosis but may be organ- and cell-specific. Hickey *et al*.^13^ reported that mutation of VHL causes Chuvash disease with pulmonary vascular remodeling, hypertension, and lung fibrosis in older mice, whereas another group found that VHL overexpression increased lung fibroblast proliferation, fibronectin and collagen abundance, and extracellular fibronectin^14^. In addition, VHL-null hearts developed fibrosis in an HIF-1α-dependent manner^15^. It is recently reported that conditional inactivation of the mouse von Hippel–Lindau tumor suppressor gene results in wide-spread hyperplastic, inflammatory and fibrotic lesions in the kidney^16^. In the liver, conditional inactivation of VHL in hepatocytes resulted in liver inflammation and hepatic steatosis^17^. When liver VHL was disrupted in mice, alcohol treatment caused marked fibrosis when compared with littermate controls^12^.

To date, little is known about the change of VHL during liver fibrogenesis, whether regulation of VHL could inhibit the progress of liver fibrosis and contribute to the antifibrogenic potential of HSCs. We have previously demonstrated that VHL overexpression inhibited the accumulation of HIF-1α and HIF-2α and angiogenesis in HCC^18^. In this study, we investigated the change of VHL expression during liver fibrosis and whether overexpression of VHL may have a therapeutic benefit to attenuate liver fibrosis and further illustrated the underlying mechanism.

## Materials and Methods

### Study Approval

All investigations of experimental animals and human subjects were reviewed and approved by the Ethics Committee the Harbin Medical University, Harbin, China. All experiments were carried out in accordance with the approved guidelines and regulations (Declaration of Helsinki). All possible efforts were made to minimize the animals’ suffering and to reduce the number of animals used. Each patient involved in this study provided written informed consent.

### Human Samples

A total of 30 matching non-tumor liver fibrosis samples (hepatitis C n = 10, alcoholic n = 10, cholestatic n = 10) were obtained from patients undergoing surgical hepatectomy or liver transplantation for HCC or hilar cholangiocarcinoma. Fragments of normal livers (n = 10) were obtained from resections of liver metastasis of colon cancer or liver hemangioma. During operation, samples were collected at least 5 cm away from the tumor border and were shown to no tumor cells by microscopy. The characteristics of the human liver sample are listed in Supplementary Table 1.

### Animal Models

Ten-week-old male C57BL/6j mice were used in all experiments. To induce liver fibrosis, two models were used. In the first model, cholestasis was induced by placing a ligature around the common bile duct. Control mice underwent sham operations, and all mice were sacrificed 3 weeks following the procedure. In the second model, mice were intraperitoneally injected twice a week with 0.5 mL/kg body wt CCl_4_ (Sigma Chemical Co., St. Louis, MO) diluted 1:3 in corn oil, while controls received an equal volume of corn oil. All mice were sacrificed 6 weeks after the first injection.

### Viral Infection

A replication-deficient serotype 5 adenoviral vector containing cDNA encoding the mouse VHL gene was constructed as described previously^18^. Expression plasmids pcDNA3 HIF-1α TM (a mutant with the triple mutation [TM] P402A/P577A/N813A to make the mouse HIF-1α protein normoxia-active) and pcDNA3 HIF-2α TM (a mutant with the triple mutation [TM] P405A/P530A/N851A to make the mouse HIF-2α protein normoxia-active) were kindly provided by Dr. Celeste Simon, University of Pennsylvania^19^. Adenoviral vectors delivering HIF-1α TM (Ad-HIF-1α TM) and HIF-2α TM (Ad-HIF-2α TM) were constructed. An Ad-Null vector was used as a negative control. Two lentiviral vectors delivering siRNAs that targeted mouse HIF-1α (LV-siHIF-1α) and HIF-2α (LV-siHIF-2α) were constructed (Supplementary Materials and Methods)^20^,^21^. *In vivo*, the adenovirus (1 × 10^9^ or 2 × 10^9^ TCID50 per mouse) and lentivirus (1 × 10^9^ IU per mouse) were injected into the tail veins in mice. Infection efficiency was confirmed with vectors encoding EGFP (Supplementary Fig. 1).

### Liver Tests

Quantitative determination of serum aspartate aminotransferase (AST) and alanine aminotransferase (ALT) levels was performed using spectrophotometric analysis (Pointe Scientific, Inc., Canton, MI).

### Histologic and Immunohistochemical Studies

The liver sections were stained with hematoxylin and eosin (H&E), Masson’s trichrome, and Sirius Red/Fast Green. Quantification of Sirius Red/Fast Green staining intensities and immunohistochemistry of liver sections are described in the Supplementary Materials and Methods. Microvessel density (MVD) was determined by counting the CD31-positive vessels in three high-power fields (x100 magnification) from areas with the highest vascularization.

### TUNEL assay

The terminal deoxynucleotidyl transferase (TdT)-mediated dUTP nick-end labeling (TUNEL) assay was performed using a commercial kit (Roche, Shanghai, China), and images were captured using a fluorescent microscope (LSM-510, Carl Zeiss Jena GmbH, Jena, Germany). TUNEL-positive nuclei were then counted in five 200× fields from each specimen.

### RNA Preparation and Real-time RT-PCR

RNA was extracted from tissues and reverse transcribed, and real-time RT-PCR was performed as previously described^22^. The primer sequences are listed in Supplementary Table 2.

### Mouse HSC Isolation and Treatment

Mouse primary HSCs were isolated and cultured as previously described^23^. Immunocytochemistry, cell viability, and apoptosis assays are described in the Supplementary Materials and Methods.

### Statistical Analysis

Data are expressed as means ± standard errors (SE) of mean. One-way analysis of variance (ANOVA) or the two-tailed Student’s t test was used where appropriate. A P value < 0.05 was considered statistically significant.

## Results

### VHL expression was inhibited in patients with liver fibrosis

**Figure 1:**
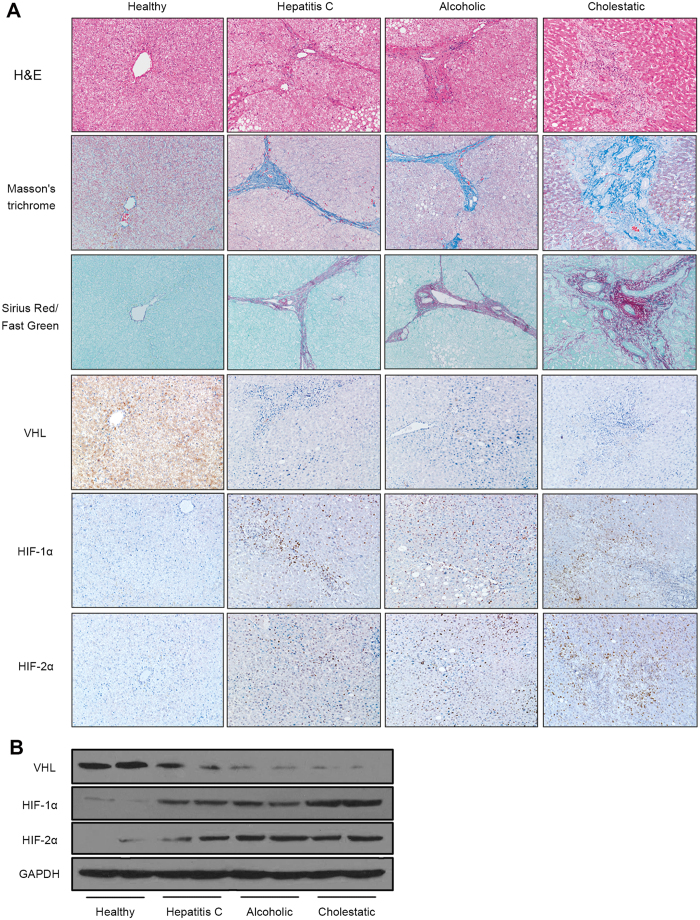
VHL expression was inhibited in patients with liver fibrosis. (**A**) Representative liver sections (original magnification, 100x) of H&E, Masson’s trichrome and Sirius Red/Fast Green staining, and immunohistochemistry for VHL, HIF-1α, and HIF-2α from healthy and cirrhotic liver sections (n = 10 for each group). (**B**) Western blot analysis of VHL, HIF-1α, and HIF-2α expression, and the gels have been run under the same experimental conditions.

As shown in Fig. 1A, marked inflammation and fibrosis were observed in fibrotic regions in livers from fibrosis patients (hepatitis C, alcoholic, cholestatic). Immunohistochemistry revealed decreased VHL expression and accumulation of HIF-1α and HIF-2α compared with samples from healthy explants (Fig. 1A). Western blot analysis showed a decrease in VHL and increases in HIF-1α and HIF-2α in patients with fibrosis compared with healthy explants (Fig. 1B). These results demonstrate that VHL expresses in human liver and is downregulated in fibrosis.

### VHL expression was inhibited in fibrosis in mice

**Figure 2:**
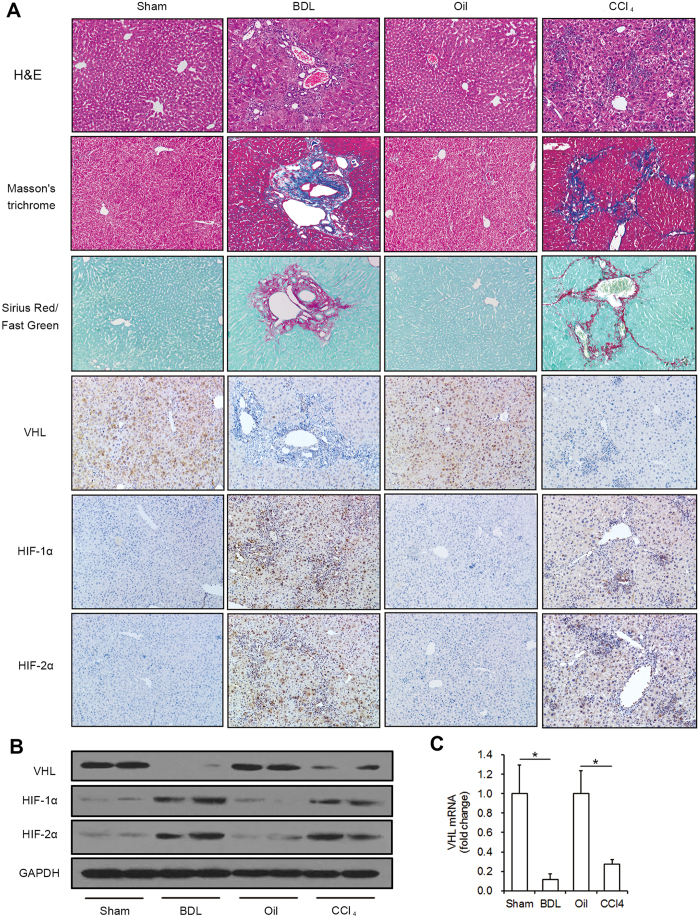
VHL expression is inhibited in fibrosis in mice. (**A**) Representative liver sections (original magnification, 100x) of H&E, Masson’s trichrome, and Sirius Red/Fast Green staining and immunohistochemistry for VHL, HIF-1α, and HIF-2α from livers of sham, BDL, oil and CCl_4_ groups (n = 10 mice for each group). (**B**) Western blot analysis of VHL, HIF-1α and HIF-2α expression, and the gels have been run under the same experimental conditions. (**C**) The Real-time RT-PCR of VHL (*p < 0.001).

To determine whether VHL also decreased during liver fibrosis in mice, we used two models, BDL and long-term CCl_4_ injection. As shown in Fig. 2A, the mouse livers developed marked inflammation and fibrosis after BDL or CCl_4_ injection. Immunohistochemistry of fibrotic regions in livers from BDL- and CCl_4_-treated mice showed a decrease in VHL expression and accumulation of HIF-1α and HIF-2α compared with livers of the control group, in accordance with our findings in human liver samples. Western blot analysis further confirmed the changes in VHL, HIF-1α, and HIF-2α in BDL- and CCl_4_-treated mice (Fig. 2B). The Real-time RT-PCR results showed the VHL mRNA significantly decreased 89% and 73% in the livers of BDL- and CCl_4_-treated mice, respectively (Fig. 2C).

### Overexpression of VHL attenuated hepatic fibrosis in mice

**Figure 3:**
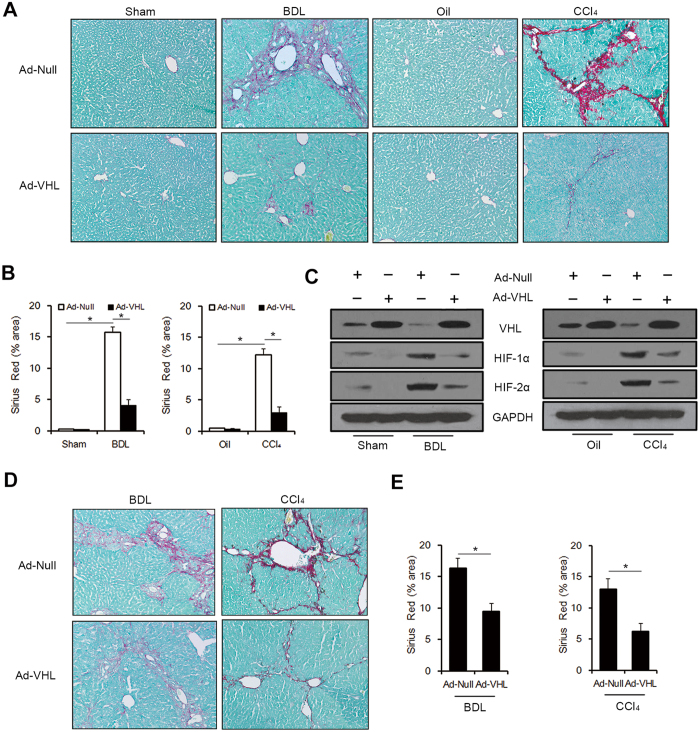
VHL overexpression attenuated hepatic fibrosis in mice. Ad-Null or Ad-VHL were injected initially in Sham, BDL, Oil or CCl_4_ group (**A**,**B** and **C**), or injected in the established liver fibrosis models (**D** and **E**). (**A**) Representative liver sections of Sirius Red/Fast Green staining (original magnification, 100x) (**B**) Quantification of Sirius Red/Fast Green staining intensities (*p < 0.001). (**C**) Western blot analysis of VHL, HIF-1α, and HIF-2α expression, and the gels have been run under the same experimental conditions. (**D**) Representative liver sections of Sirius Red/Fast Green staining (original magnification, 100 x) (**E**) Quantification of Sirius Red/Fast Green staining intensities (*p < 0.001).

To confirm the role of VHL in liver fibrogenesis, we overexpressed VHL by injecting Ad-VHL in mice. In the BDL groups (n = 10 for each group), mice were injected in the tail vein with 1 × 10^9^ TCID50 of Ad-Null or Ad-VHL suspended in 100 μl of PBS after sham or BDL, and sacrificed 3 weeks later. In CCl_4_ groups (n = 10 for each group), mice were injected intraperitoneally with Oil or CCl_4_, injected in the tail vein with 1 × 10^9^ TCID50 of Ad-Null or Ad-VHL at days 0 and 21, and sacrificed 6 weeks after the initial injection. The results showed that injection of Ad-VHL led to reduced histological fibrosis in BDL- and CCl_4_-treated mice compared with injection of Ad-Null, as seen by Sirius Red/Fast Green staining (Fig. 3A and B). Injection of Ad-VHL resulted in upregulation of VHL and downregulation of HIF-1α and HIF-2α in BDL- and CCl_4_-treated mice, as seen by Western blot analysis (Fig. 3C). Consistent with HIF-α accumulation in fibrotic livers of BDL- and CCl4-treated mice, an increase in glucose transporter 1 (Glut1), lactate dehydrogenase A (LDHA), pyruvate dehydrogenase kinase 1 (PDK1) and erythropoietin (EPO), four classic HIF-α target genes, were observed (Table 1). The up-regulations of above genes were inhibited by VHL overexpression, except LDHA in BDL groups (Table 1). In addition, VHL overexpression also led to reduced expression of genes involved in fibrogenesis, such as collagen-1α, transforming growth factor-β1 (TGF-β1), tissue inhibitor of metallopeptidase-1 (TIMP-1), plasminogen activator inhibitor-1 (PAI-1), and platelet-derived growth factor-B (PDGF-B) (Table 1). Then, we injected 1 × 10^9^ TCID50 of Ad-Null or Ad-VHL 2 weeks after BDL and 4 weeks after CCl_4_ initial injection, and the mice were sacrificed 1 week or 2 weeks respectively. The results showed that injection of Ad-VHL still led to reduced histological fibrosis in BDL- and CCl_4_-treated mice compared with injection of Ad-Null (Fig. 3D and E), which indicated that VHL overexpression can be therapeutic in mouse models of established liver fibrosis. Altogether, our results suggest that VHL plays a crucial role in liver fibrosis.

### Overexpression of VHL attenuated liver inflammation, apoptosis, and angiogenesis

**Figure 4:**
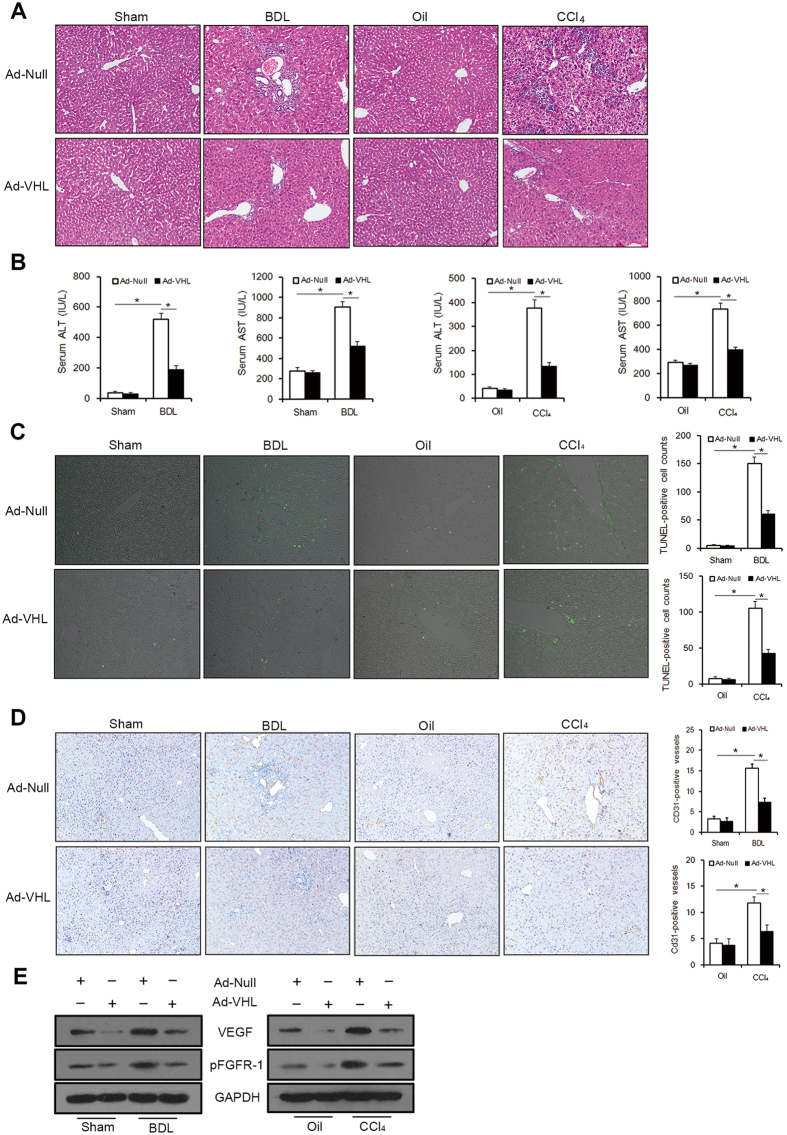
VHL overexpression attenuated liver inflammation, apoptosis, and angiogenesis. 1 × 10^9^ TCID50 of Ad-Null or Ad-VHL were injected in BDL or CCl_4_ groups. (**A**) Representative liver sections of H&E staining (original magnification, 100x). (**B**) Serum ALT and AST levels (*p < 0.001). (**C**) Representative liver sections stained with the TUNEL reagent to visualize apoptotic cells (original magnification, 200x), and TUNEL-positive nuclei were counted (*p < 0.001). (**D**) Representative liver sections were stained for CD31 to visualize microvessels (original magnification, 100x), which were counted (*p < 0.001). (**E**) Western blot analysis of VEGF and pFGFR-1 expression and the gels have been run under the same experimental conditions.

To examine the mechanism of VHL inhibiting hepatic fibrosis, we investigated liver inflammation, apoptosis, and angiogenesis. As expected, severe inflammation was observed in BDL- and CCl_4_-treated mice (Fig. 4A). By contrast, hepatocellular injury and inflammatory cell-infiltration were milder in Ad-VHL-treated mice compared with Ad-Null-treated mice, as shown in H&E staining (Fig. 4A). Serum ALT and AST values, an index of hepatocyte injury, were also significantly lower in Ad-VHL-treated mice versus in Ad-Null-treated mice (Fig. 4B). Furthermore, VHL overexpression led to reduced expression of genes involved in inflammation, such as tumor necrosis factor-α (TNF-α), monocyte chemoattractant protein-1 (MCP-1), and macrophage inflammatory proteins (MIP)-1β and MIP-2 (Table 1). Liver cell apoptosis (as measured by TUNEL-positive cells) was significantly reduced in the Ad-VHL group compared with the Ad-Null group (Fig. 4C). These data implicate a protective role for VHL in liver injury and apoptosis. Furthermore, staining for CD31, an important marker for neomicrovessels, was enhanced in the liver sinusoid in BDL- and CCl_4_-treated mice. CD31-positive staining in the sinusoid was diminished with Ad-VHL administration (Fig. 4D). Separate from the angiogenesis marker CD31, the angiogenic factors vascular endothelial growth factor (VEGF) and phosphorylated fibroblast growth factor receptor-1 (pFGFR-1) were downregulated in fibrotic livers after Ad-VHL treatment (Fig. 4E).

### Overexpression of VHL attenuated hepatic fibrosis via HIF-1α and HIF-2α

**Figure 5:**
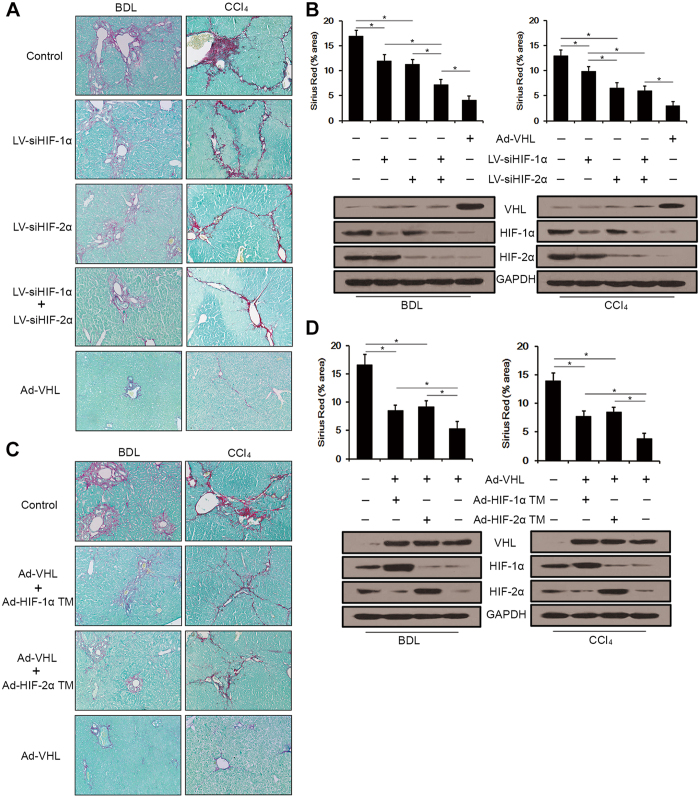
VHL overexpression attenuated hepatic fibrosis via HIF-1α and HIF-2α. HIF-1α and HIF-2α were silenced by lentivirus vectors encoding siRNAs. (**A**) Representative liver sections of Sirius Red/Fast Green staining (original magnification, 100x). (**B**) Sirius Red/Fast Green staining intensities were quantified (*p < 0.001), and expressions of VHL, HIF-1α and HIF-2α were measured by Western blot analysis, and the gels have been run under the same experimental conditions. VHL, HIF-1α TM, and HIF-2α TM were overexpressed from adenovirus vectors. (**C**) Representative liver sections of Sirius Red/Fast Green staining (original magnification, 100x). (**D**) Sirius Red/Fast Green staining intensities were quantified (*p < 0.001), and expressions of VHL, HIF-1α and HIF-2α were measured by Western blot analysis, and the gels have been run under the same experimental conditions.

To confirm the involvement of HIF-1α and HIF-2α in the regulation of liver fibrosis in BDL- and CCl_4_-treated mice, we silenced HIF-1α, HIF-2α, or both *in vivo*. In BDL groups (n = 9 for LV-siHIF-1α + LV-siHIF-2α group, n = 10 for the other groups), mice were injected into the tail vein with 1 × 10^9^ IU control lentivirus, 0.5 × 10^9^ IU control lentivirus + 0.5 × 10^9^ IU LV-siHIF-1α, 0.5 × 10^9^ IU control lentivirus + 0.5 × 10^9^ IU LV-siHIF-2α, 0.5 × 10^9^ IU LV-siHIF-1α + 0.5 × 10^9^ IU LV-siHIF-2α or 1 × 10^9^ TCID50 Ad-VHL after BDL and were sacrificed 3 weeks later. In CCl_4_ groups (n = 10 for each group), CCl_4_-treated mice were injected into the tail vein with the same vectors as the BDL groups on days 0 and 21. Mice were sacrificed 6 weeks after the initial injection. Sirius Red/Fast Green staining showed that silencing HIF-1α or HIF-2α separately inhibited liver fibrosis compared with the control group (Fig. 5A and B). In the BDL groups, silencing HIF-1α plus HIF-2α achieved a greater attenuation of fibrosis than silencing HIF-1α or HIF-2α alone, and the difference between LV-siHIF-1α and LV-siHIF-2α treatment did not reach significance (p = 0.149). In the CCl_4_ groups, the effect of HIF-2α silencing in attenuating liver fibrosis was significantly higher compared with the effect of silencing HIF-1α, however, silencing HIF-1α plus HIF-2α did not achieve a greater attenuation of fibrosis than silencing HIF-2α alone (p = 0.280). In both the BDL and CCl_4_ groups, silencing HIF-1α plus HIF-2α was significantly less successful at inhibiting fibrosis compared with overexpression of VHL (Fig. 5A and B). Silencing of HIF-1α or HIF-2α was confirmed by Western blot analysis (Fig. 5B). To extend our findings, we next tested whether VHL-mediated inhibition of liver fibrosis was HIF-1α- or HIF-2α-dependent. Using adenoviral vectors, we overexpressed HIF-1α TM or HIF-2α TM (mutant HIFs that cannot be bound by VHL and degraded) in Ad-VHL-treated mice. In BDL groups (n = 10 for each group), mice were injected into the tail vein with 2 × 10^9^ TCID50 Ad-Null, 1 × 10^9^ TCID50 Ad-VHL + 1 × 10^9^ TCID50 Ad- HIF-1α TM, 1 × 10^9^ TCID50 of Ad-VHL + 1 × 10^9^ TCID50 Ad- HIF-2α TM or 1 × 10^9^ TCID50 of Ad-Null + 1 × 10^9^ TCID50 Ad-VHL after BDL and were sacrificed 3 weeks later. In CCl_4_ groups (n = 9 for Ad-VHL group, n = 10 for the other groups), CCl_4_-treated mice were injected into the tail vein with the same adenoviral vector as the BDL groups on days 0 and 21. All mice were sacrificed 6 weeks after initial injection. As shown in Fig. 5C and D, the overexpression of either VHL and HIF-1α TM or HIF-2α TM attenuated fibrosis compared to Ad-Null-injected mice, but overexpression of VHL alone attenuated fibrosis most effectively. There was no significant difference between the overexpression of VHL and HIF-1α TM and the overexpression of VHL and HIF-2α TM (p = 0.315 for BDL groups, and p = 0.130 for CCl_4_ groups). Overexpression of VHL, HIF-1α TM, and HIF-2α TM was confirmed by Western blot analysis (Fig. 5D). Real-time RT-PCR was used to determine the impact of HIF-α TM overexpression on fibrogenic and HIF-α target genes. As shown in Supplementary Table 3, overexpression of Ad-HIF-1α TM in Ad-VHL-injected mice resulted in the upregulation of Collagen-1, TGF-β1, TIMP-1, PAI-1, Glut1, LDHA and PDK1 in BDL- and CCl_4_-treated mice, while overexpression of Ad-HIF-2α TM in Ad-VHL-injected mice resulted in the upregulation of Collagen-1, PDGF-B and EPO in BDL-treated mice, and Collagen-1, TGF-β1, PDGF-B and EPO in CCl_4_-treated mice.

### Role of VHL in HSC activation, proliferation, and apoptosis

**Figure 6:**
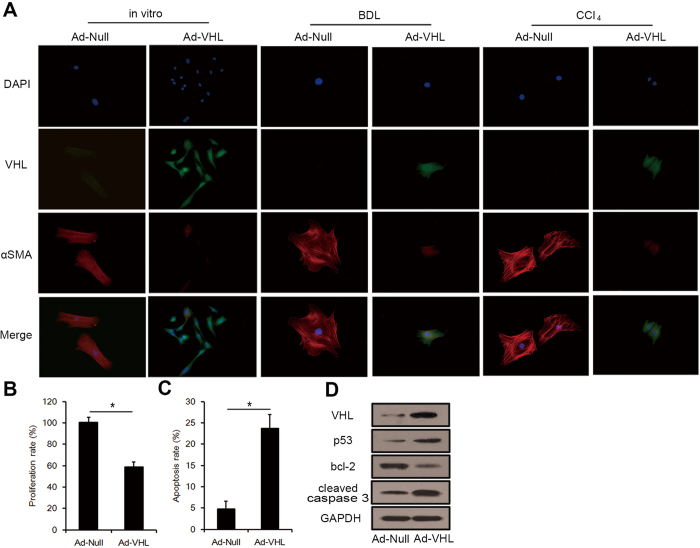
Effects of VHL on HSC activation, proliferation, and apoptosis. (**A**) Immunocytochemistry for VHL (green fluorescence) and αSMA (red fluorescence) in HSCs. (**B**) HSC proliferation was measured (*p < 0.001). (**C**) HSC apoptosis was detected. (*p < 0.001). (**D**) Western blot analysis of p53, bcl-2 and cleaved caspase 3, and the gels have been run under the same experimental conditions.

To characterize the effect of VHL on HSC activation, we examined VHL expression in primary HSCs isolated from mice. During *in vitro* activation of primary HSCs on a plastic surface, VHL levels decreased gradually after 4 or 7 days in culture compared to quiescent HSCs (day 1), an effect that correlated with HSC activation as measured by induction of αSMA expression (Supplementary Fig. 3). The decreased expression of VHL in activated HSCs (day 7) was further confirmed by Real-time RT-PCR and Western blot analysis and this expression is concomitant with HIF-1α and HIF-2α accumulation (Supplementary Fig. 4). Because VHL inhibited liver fibrosis *in vivo*, we hypothesized that VHL overexpression could inhibit HSC activation. To test this theory, primary HSCs isolated from normal livers were infected with Ad-Null or Ad-VHL (at a multiplicity of infection [MOI] of 100) and maintained in culture for 7 days. The results showed that infection of Ad-VHL inhibited intracellular αSMA expression and HSC activation. In addition, we isolated primary HSCs from BDL- and CCl_4_-treated mice. In BDL groups, mice were injected into the tail vein with 1 × 10^9^ TCID50 of Ad-Null or Ad-VHL after BDL and sacrificed 3 weeks later to isolate primary HSCs. In CCl_4_ groups, mice were injected in the tail vein with 1 × 10^9^ TCID50 of Ad-Null or Ad-VHL at days 0 and 21, and sacrificed 6 weeks after the initial injection to isolate primary HSCs. Then, HSCs isolated from BDL- and CCl_4_-treated mice were cultured for 24 h on a plastic surface. As shown in Fig. 6A, VHL levels were extremely low in activated HSCs from BDL- and CCl_4_-treated mice injected with Ad-Null. A more important finding was that Ad-VHL injection upregulated the expression of VHL in HSCs *in vivo*, and along with enhanced intracellular VHL expression, intracellular αSMA expression and HSC activation were inhibited by Ad-VHL infection compared with Ad-Null (Fig. 6A). Hence, an inhibitory role for VHL in modulating αSMA deposition and HSC activation was established. Following this result, primary activated HSCs (day 7) were treated with Ad-Null or Ad-VHL (MOI of 100) for 72 h. The results showed that VHL overexpression significantly inhibited the proliferation of activated HSCs (Fig. 6B). Furthermore, Annexin V-fluorescein isothiocyanate (FITC) and propidium iodide (PI) was used to detect apoptosis cells. Ad-VHL significantly increased the level of apoptosis in activated HSCs (day 7) compared with HSCs treated with Ad-Null after 48 h treatment (Fig. 6C). To investigate whether pro-apoptotic signaling pathways were activated following Ad-VHL infection, lysates of HSCs that had been treated for 24 h with Ad-VHL were analyzed by Western blot for bcl-2, p53, and caspase-3. As shown in Fig. 6D, Ad-VHL treatment resulted in reduced bcl-2 protein levels, increased p53 expression, and caspase-3 cleavage.

## Discussion

Several studies have illuminated the role of HIF-α in the pathogenesis of chronic liver diseases that cause liver fibrosis. Hepatitis B virus X protein was found to induce HIF-1α expression and stability^24^. Hepatitis C virus was reported to stabilize HIF-1α *in vitro*^25^. Later, stabilization of HIF-1α was noted in liver biopsy specimens obtained from patients with chronic hepatitis C^26^. In hepatocyte cell lines expressing hepatitis E virus, open reading frame protein 3 correlated with the increased expression and DNA-binding activity of HIF-1α^27^. Recent work has demonstrated that chronic alcohol administration increased HIF-1α activation in the liver and increased hepatic steatosis in an HIF-1α-dependent manner^28^. For the first time, our present study has demonstrated that VHL expression is dramatically inhibited in human hepatitis C, alcoholic, and cholestatic cirrhotic livers compared with healthy liver samples and that this expression is concomitant with HIF-1α and HIF-2α accumulation. In BDL- and CCl_4_-treated mice, we further demonstrated that VHL, HIF-1α, and HIF-2α protein levels are in accordance with the results from human liver samples. Moon *et al*. found that HIF-1α was activated in the livers of mice subjected to BDL before significant fibrosis is normally present. Interestingly, although HIF-1α activation was detected in areas of bile duct proliferation, hypoxia in this region was not detected by pimonidazole immunostaining^10^. Our findings demonstrated that HIF-1α and HIF-2α accumulation in chronic liver diseases^24^,^25^,^26^,^27^,^28^ or in normoxia regions of the liver^10^ might be, at least in part, attributed to diminished VHL expression. These findings underscore the vital role of VHL in liver fibrosis.

The specific underlying liver disease is thought to determine the cell types that contribute to the myofibroblast pool, with profound differences between cholestatic and toxic liver diseases. To allow more general conclusions, we used two different animal models, cholestatic fibrosis and toxic fibrosis. In both models, we found that VHL overexpression attenuated liver fibrogenesis and inhibited HIF-1α and HIF-2α accumulation and the expressions of their target genes. We found VHL overexpression might be therapeutic in mouse models of established liver fibrosis, but it is also possible that it inhibits the progression of liver fibrosis in the last 1 or 2 weeks of the experimental models. TGF-β1 is a major profibrogenic cytokine and plays a vital role in the activation of HSCs. Particularly, TGF-β1 induces phenotypic transdifferentiation of HSCs and synthesis of ECM^29^. PDGF-B is the most potent stimulus for HSCs proliferation and migration and contributes to the perpetuation of liver fibrosis^30^. It has been reported that VHL is involved in the negative regulation of both TGF-β1 and PDGF-B in wound healing^31^. Here, we found that VHL overexpression downregulated both TGF-β1 and PDGF-B during liver fibrogenesis. In healthy livers, homeostasis of the extracellular matrix is sustained by matrix metalloproteinases (MMPs) and their specific inhibitors, TIMPs. Anti-TIMP-1 antibodies led to a reduction of hydroxyproline content and αSMA staining^32^ and TIMP-1 antisense oligonucleotides could induce hepatic fibrolysis^33^, suggesting that TIMP-1 may represent an attractive therapeutic target for fibrogenesis and fibrolysis. In this study, we observed enhanced expression of TIMP-1 in two mouse models, provoked via CCl_4_ injection and bile duct ligation, similar to the rat models^34^, and also found that VHL overexpression inhibited TIMP-1 and attenuated liver fibrosis.

Furthermore, our results showed that VHL overexpression also inhibited liver inflammation, apoptosis, and angiogenesis, all of which contribute to deteriorating liver fibrogenesis. The role of neovascularization in the liver during fibrosis progression is still controversial. In most of the published data, aberrant angiogenesis is clearly implicated in hepatic fibrosis progression and is considered a major determinant of hepatic dysfunction and irreversibility in cirrhosis^35^. However, Patsenker *et al*. found that antiangiogenic therapy via pharmacological inhibition of αvβ3 integrin promoted fibrosis progression in HSCs^36^. Our previous work illustrated the inhibitory effect of VHL on microvessels in HCC^18^. In the present study, VHL overexpression resulted in reduced microvessels in both cholestatic and toxic fibrotic livers and anti-angiogenesis is concomitant with inhibition of live fibrosis. VEGF, a vital angiogenic factor, had a fibrogenic effect through multiple mechanisms^37^. A recent finding indicated a dual action of VEGF on stimulating fibrosis. Anti-VEGF antibodies that selectively block VEGF impaired BDL-induced fibrogenesis, whereas tissue repair was impaired by VEGF inhibition, indicating that VEGF was required for hepatic tissue repair and fibrosis resolution^38^. Fibroblast growth factor 2 (FGF-2) stimulates angiogenesis synergistically with VEGF but with different effects on vessel size and function. FGF-2 also acts as a mitogen for HSCs and overexpression of its receptor (FGFR-1) has been reported in human liver myofibroblasts^39^. Recently, time-dependent upregulation and activation of FGFR-1 after BDL was reported, and FGFR-1-mediated CXCR4 upregulation and CXCR7 suppression in liver counterbalanced the pro-regenerative function of liver sinusoidal endothelial cells, leading to fibrosis^40^. In the present study, we found that the expressions of both VEGF and pFGFP were dramatically induced by BDL and CCl_4_ injection. Meanwhile, VEGF and pFGFR-1 levels were both significantly inhibited by VHL overexpression. By targeting VEGF and pFGFR-1, VHL inhibited neovascularization in the liver during fibrosis, which might account for the effect of VHL on liver fibrosis.

It has been reported that collagen I, αSMA, PDGF-B, and PAI-1 were significantly reduced in HIF-1α-deficient mice compared with control mice subjected to BDL, indicating that HIF-1α is a critical regulator of profibrotic mediator production during liver fibrosis^10^. More recent work has argued for a dominant role of HIF-2α in regulating hepatic fibrogenesis in the setting of steatohepatitis. The increase in fibrosis observed in VHL-disrupted mice on an alcohol diet was completely lost in a VHL and HIF-2α double knockout, but not in a VHL and HIF-1α double knockout^12^. Our results showed that silencing HIF-1α or HIF-2α alone inhibited liver fibrosis, but silencing HIF-1α plus HIF-2α achieved a further attenuation in fibrosis in BDL groups. The effect of HIF-2α silencing in attenuating liver fibrosis was significant compared with HIF-1α silencing in CCl_4_ groups, indicating that HIF-2α may play a dominant role in toxic liver fibrosis. However, silencing HIF-1α alone also attenuated liver fibrosis, so the role of HIF-1α cannot be ignored. Our results indicate that HIF-1α and HIF-2α play different roles depending on the aetiology of liver fibrosis. Interestingly, when VHL and HIF-1α or HIF-2α were both overexpressed, HIF-1α and HIF-2α were equally effective at preventing the inhibitory effect of VHL on liver fibrosis, and the dominant effect of HIF-2α in toxic liver fibrosis did not exist. In addition, silencing HIF-1α plus HIF-2α was significantly weaker at inhibiting fibrosis compared with VHL overexpression, indicating that there may be an HIF-1α- and HIF-2α-independent pathway for VHL to attenuate liver fibrosis.

To dissect whether VHL could condition the behavior of HSCs, VHL expression in primary HSCs was measured and found to be dramatically inhibited, not only in self-activated HSCs *in vitro* but also in activated HSCs *in vivo*. We further overexpressed VHL *in vitro* and *in vivo* to illustrate its inhibitory effect on HSC activation and showed that proliferation of activated HSCs is inhibited by VHL *in vitro*. The removal of hepatic myofibroblasts by apoptosis is considered a key mechanism in reducing the number of activated myofibroblasts and allowing the liver to return to its normal architecture. We assessed the effect of VHL on apoptosis of activated HSCs and found a dual benefit; VHL significantly induces apoptosis of activated HSCs and inhibits hepatic cell apoptosis *in vivo*.

In conclusion, our results revealed that VHL is expressed in the liver and decreases upon the onset of chronic liver injury, concomitant with HIF-1α and HIF-2α accumulation. Furthermore, VHL overexpression attenuates liver fibrosis through a HIF-α-dependent pathway and inactivates HSCs both *in vitro* and *in vivo*. Thus, VHL may be considered a new target to prevent the development and progression of liver fibrosis.

## Additional Information

**How to cite this article**: Wang, J. *et al*. Reduction of hepatic fibrosis by overexpression of von Hippel–Lindau protein in experimental models of chronic liver disease. *Sci. Rep.* **7**, 41038; doi: 10.1038/srep41038 (2017).

**Publisher's note:** Springer Nature remains neutral with regard to jurisdictional claims in published maps and institutional affiliations.

## Supplementary Material

Supplementary Document

## Figures and Tables

**Table 1:** Transcript levels of genes related to fibrosis and inflammation in mice.

Target gene	Sham		BDL		Oil		CCl_4_	
	Ad-Null	Ad-VHL	Ad-Null	Ad-VHL	Ad-Null	Ad-VHL	Ad-Null	Ad-VHL
Glut1	1.00 ± 0.31	0.39 ± 0.19	39.58 ± 7.52*	12.45 ± 3.88^•^	1.00 ± 0.25	0.51 ± 0.20	6.78 ± 1.93*	0.63 ± 0.35^•^
LDHA	1.00 ± 0.20	0.99 ± 0.33	9.26 ± 2.54*	7.79 ± 2.51	1.00 ± 0.34	0.85 ± 0.34	6.35 ± 1.72*	1.52 ± 0.43^•^
PDK1	1.00 ± 0.26	0.94 ± 0.26	9.05 ± 3.07*	2.48 ± 0.57^•^	1.00 ± 0.23	0.80 ± 0.25	8.00 ± 1.78*	3.00 ± 0.49^•^
EPO	1.00 ± 0.26	0.33 ± 0.11	40.76 ± 6.18*	17.50 ± 4.02^•^	1.00 ± 0.26	0.42 ± 0.07	28.67 ± 7.02*	3.04 ± 0.84^•^
Collagen-1α	1.00 ± 0.32	0.48 ± 0.11	21.99 ± 1.61*	7.01 ± 0.63^•^	1.00 ± 0.19	0.80 ± 0.12	14.79 ± 1.54*	5.30 ± 0.75^•^
TGF-β1	1.00 ± 0.29	0.47 ± 0.11	14.36 ± 2.50*	7.65 ± 1.62^•^	1.00 ± 0.24	0.80 ± 0.20	6.38 ± 1.08*	2.77 ± 0.64^•^
TIMP-1	1.00 ± 0.32	0.73 ± 0.23	5.14 ± 0.57*	1.73 ± 0.34^•^	1.00 ± 0.32	0.93 ± 0.10	4.28 ± 0.58*	1.51 ± 0.27^•^
PAI-1	1.00 ± 0.25	0.72 ± 0.21	9.11 ± 1.54*	4.73 ± 0.95^•^	1.00 ± 0.22	0.78 ± 0.17	6.27 ± 0.74*	2.60 ± 0.54^•^
PDGF-B	1.00 ± 0.20	0.77 ± 0.22	7.70 ± 1.20*	2.87 ± 0.56^•^	1.00 ± 0.27	0.98 ± 0.17	5.30 ± 0.67*	1.97 ± 0.53^•^
TNF-α	1.00 ± 0.21	0.60 ± 0.12	15.20 ± 2.10*	6.84 ± 1.58^•^	1.00 ± 0.27	0.79 ± 0.15	4.46 ± 0.60*	2.02 ± 0.39^•^
MCP-1	1.00 ± 0.27	1.07 ± 0.24	7.14 ± 1.09*	2.31 ± 0.41^•^	1.00 ± 0.21	0.63 ± 0.12	4.31 ± 0.67*	1.31 ± 0.22^•^
MIP-1β	1.00 ± 0.30	0.80 ± 0.31	11.69 ± 1.51*	4.31 ± 0.95^•^	1.00 ± 0.20	0.66 ± 0.18	6.17 ± 0.94*	2.84 ± 0.38^•^
MIP-2	1.00 ± 0.26	0.39 ± 0.08	8.81 ± 1.17*	3.56 ± 0.71^•^	1.00 ± 0.21	0.70 ± 0.16	7.73 ± 0.91*	2.64 ± 0.52^•^

*p < 0.001 vs Ad-Null + sham or Ad-Null + Oil, ^•^p < 0.001 vs Ad-Null + BDL or Ad-Null + CCl_4_.
