# Supplementary material for: Reduction of hepatic fibrosis by overexpression of von Hippel–Lindau protein in experimental models of chronic liver disease
Source: Sci Rep. 2017 Jan 23;7:41038. doi: 10.1038/srep41038 (PMC5253623; doi:10.1038/srep41038)
Supplement: Supplementary Document [file srep41038-s1.doc]

**Supplementary material**

**Title:** Reduction of hepatic fibrosis by overexpression of von Hippel–Lindau protein in experimental models of chronic liver disease

***Authors:***

Jizhou Wang^1*^, Zhaoyang Lu^1*^, Zhilin Xu^2*^, Pei Tian^3^, Hui Miao ^2^, Shangha Pan^1^, Ruipeng Song^1^, Xueying Sun^1,4^, Baolei Zhao^1^, Dawei Wang^1^, Yong Ma^1^, Xuan Song^1^, Shugeng Zhang^1^, Lianxin Liu^1#^, Hongchi Jiang^1#^

^1^Key Laboratory of Hepatosplenic Surgery, Department of Hepatic Surgery, The First Affiliated Hospital of Harbin Medical University, Harbin 150001, China.

^2^Department of Pediatric Surgery, The First Affiliated Hospital of Harbin Medical University, Harbin 150001, China.

^3^Department of Ophthalmology, The First Affiliated Hospital of Harbin Medical University, Harbin 150001, China.

^4^ Department of Molecular Medicine and Pathology, School of Medical Sciences, University of Auckland, Auckland 1023, New Zealand

*These authors contributed equally to this work.

# Corresponding authors.

Address: Key Laboratory of Hepatosplenic Surgery, Department of General Surgery, The First Affiliated Hospital of Harbin Medical University, Harbin, 150001, China. Tel.:+86-0451-85552358. Fax: +86-0451-85552358. Email address: [jianghc@vip.163.com](mailto:jianghc@vip.163.com) (H Jiang).

Address: Key Laboratory of Hepatosplenic Surgery, Department of General Surgery, The First Affiliated Hospital of Harbin Medical University, Harbin, 150001, China. Tel.:+86-0451-85552358. Fax: +86-0451-85552358. Email address: [liulianxin@medmail.com.cn](mailto:liulianxin@medmail.com.cn) (L Liu).

**Materials and Methods**

*Quantification of fibrosis*

To evaluate the severity of fibrosis, a standard 1063×797-pixel region of interest (ROI) was taken from each sample by a 'blinded' pathologist and analyzed using ImageJ software (National Institutes of Health, Bethesda, MD), which provided the percentage of fibrosis in each ROI using Sirius Red/Fast Green.

*Immunohistochemistry*

Antigen retrieval was performed with citrate buffer, followed by blocking with 0.6% H_2_O_2_ and 2% goat serum. Antibodies against VHL (Santa Cruz Biotechnology, CA, USA), HIF-1α, HIF-2α and CD31 (Abcam Inc., MA, USA) were applied overnight at 1:200 dilution, followed by secondary antibody for 45 min at room temperature and diaminobenzidine detection. Sections were counterstained, mounted, and examined by microscopy.

*Immunocytochemistry*

Immunocytochemistry was performed on primary HSCs by fixing cells in 10% formalin for 30 minutes, washing in PBS, and then incubating with an FITC-conjugated αSMA antibody and VHL antibody for 2 hours. Cells were also stained with 4',6-diamidino-2-phenylindole (DAPI) to mark nuclei and were viewed by laser scanning confocal microscopy (LSM-510, Carl Zeiss Jena GmbH, Jena, Germany).

*Cell viability assay*

The cells were seeded onto a 96-well plate (3 ×10^3^/well) and cultured overnight. The culture medium was replaced with fetal calf serum-free media containing Ad-Null or Ad-VHL at an MOI of 100 for 90 min, followed by replacement of the culture media with 10% fetal calf serum-supplemented media. The cells were cultured for 72 h, and cell viability was measured with a Cell Counting Kit-8 (CCK-8) kit (Dojindo Molecular Technologies, Gaithersburg, MD, USA). Untreated cells served as controls. The cell viability index was calculated according to the following formula: (experimental OD value/control OD value)×100%.

*Apoptosis assay*

Cells were washed with PBS, resuspended in binding buffer, and incubated with Annexin V and PI for 15 min according to the manufacturer’s instructions (BD Biosciences, San Jose, California, USA). The cells were analyzed in a cytometer to measure the apoptosis rate (expressed as a percentage).

*siRNAs targeting HIF-αs*

The mouse HIF-1α siRNA target sequences were 5’-AGAGGUGGAUAUGUCUGGG-3’ (sense) and 5’-CCCAGACAUAUCCACCUCU-3’ (antisense), and the mouse HIF-2α siRNA target sequences were 5’- CUCAGUUACAGCCACAUCGUCACUG-3’ (sense) and 5'-CAGUGACGAUGUGGCUGUAACUGAG-3' (antisense). A lentiviral vector expressing a scrambled RNA was used as the control.

*Infection efficiencies of Ad-EGFP and LV-EGFP in vivo*

Ten-week-old male C57BL/6j mice were injected into the tail veins with 1×10^9^ TCID50 Ad-EGFP or 0.5×10^9^ IU LV-EGFP. Two days later, the mice were sacrificed, and their livers were harvested and cut into 5 μm frozen sections that were viewed by laser scanning confocal microscopy (LSM-510, Carl Zeiss Jena GmbH, Jena, Germany). EGFP expression is shown in Supplementary Fig. 1.

*Western Blotting*

Whole cell or tissue extracts were prepared using RIPA buffer. The protein amount loaded in the gel was 50-80μg. After electrophoresis, proteins were electroeluted at 120 volts onto a polyvinylidenedifluoride (PVDF) membrane (Invitrogen). Indicated primary antibodies were used at 1/2000-1/3000 dilution to detect expressions of VHL, HIF-1α, HIF-2α, VEGF, pFGFR-1, p53, bcl-2, cleaved caspase 3 and GAPDH, as follows: VHL (Santa Cruz sc-5575), HIF-1α (Abcam ab2185 or Novus NB100-479), HIF-2α (Abcam ab199 or Novus NB100-122), VEGF (Novus NB100-2381), pFGFR-1 (Abcam ab194527), p53 (Novus NB100-92601), bcl-2 (Cell Signaling Technology #3498), cleaved caspase 3 (Cell Signaling Technology #9664) and GAPDH (Santa Cruz sc-25778). Protein bands were visualized using an enhanced chemiluminescence assay kit (SuperSignal Pierce Biotechnology). The Western blotting analysis was repeated at least three times.

| **Characteristics** | **healthy** | **Hepatitis C** | **Alcoholic** | **Cholestatic** |
| --- | --- | --- | --- | --- |
| **n** | 10 | 10 | 10 | 10 |
| **sex** | 6 (male) | 6 (male) | 10 (male) | 7 (male) |
| **Mean age (minimum-maximum)** | 53 (37-68) | 56 (46-64) | 58 (49-65) | 53 (41-64) |
| **Primary disease for operation** | Liver metastasis of colon cancer (3)  Liver hemangioma (7) | HCC (10) | HCC (10) | Hilar cholangiocarcinoma (10 ) |
| **Mean ALT level (minimum-maximum) U/L** | 25 (9-48) | 35 (17-51) | 32 (19-55) | 82 (58-110) |
| **Mean AST level (minimum-maximum) U/L** | 28 (16-45) | 39 (24-57) | 38 (25-61) | 87 (61-130) |
| **Mean total bilirubin level (minimum-maximum) μmol/L** | 17.9 (8.3-29.8) | 20.7 (9.7-33.5) | 19.3 (10.5-26.7) | 164.3 (89-254) |
| **Mean serum albumin level (minimum-maximum) g/L** | 41.4 (31.7-50.1) | 40.3 (29.8-50.1) | 36.8 (31.5-44.5) | 38.0 (33.6-44.5) |
| **Mean primary tumor size (minimum-maximum) cm** | 7.4 (4.8-12.1) | 6.0 (3.8-8.7) | 5.3 (3.5-7.6) | 7.2 (5.6-10.5) |
| **Number of tumors** | 1 | 1 | 1 | 1 |
| **Treatment** | Partial hepatectomy (7)  Partial hepatectomy and colectomy (3) | Partial hepatectomy (8)  Liver transplantation (2) | Partial hepatectomy (9)  Liver transplantation(1) | Partial hepatectomy and hilar biliary excision (10) |

**Supplementary table 1. The characteristics of human liver samples**

**Supplementary table 2. Primer sets for real-time RT-PCR**

| **Target gene** | **Sense primer** | **Antisense primer** |
| --- | --- | --- |
| **VHL** | CAGCTACCGAGGTCATCTTTG | CTGTCCATCGACATTGAGGGA |
| **TGF****-β1** | GTGTGGAGCAACATGTGGAACTCTA | TTGGTTCAGCCACTGCCGTA |
| **TIMP-1** | CGAATCAACGAGACCACCTT | GTAGTCCTCAGAGCCCACGA |
| **MMP-9** | CAAATTCTTCTGGCGTGTGA | CGGTTGAAGCAAAGAAGGAG |
| **TNF-α** | AAGCCTGTAGCCCACGTCGTA | GGCACCACTAGTTGGTTGTCTTTG |
| **PAI-1** | TCAGCCCTTGCTTGCCTCAT | GCATAGCCAGCACCGAGGA |
| **PDGF-B** | TGAAATGCTGAGCGACCAC | GGGTCACTACTGTCTCACA |
| **MCP-1** | CTGAAGCCA GCTCTCTCTTCCT | CAGGCCCAGAAGCATGACA |
| **MIP-1β** | ATGAAGCTCTGCGTGTCTGCCCTCT | TCAGTTCAACTCCAAGTCACTCATG |
| **MIP-2** | CCACCAACCACCAGGCTAC | GCTTCAGGGTCAAGGGCAAA |
| **LDHA** | GGATGAGCTTGCCCTTGTTGA | GACCAGCTTGGAGTTCGCAGTTA |
| **Glut1** | CCA TGT ATG TGG GAG AGG TGT | TTG CCC ATG ATG GAG TCT AAG |
| **PDK1** | TTACTCAGTGGAACACCGCC | GTTTATCCCCCGATTCAGGT |
| **EPO** | CATCTGCGACAGTCGAGTTCTG | CACAACCCATCGTGACATTTTC |
| **GAPDH** | CATGGC CTTCCGTGTTCCTA | CCTGC TTCACCACCTTCTTGAT |

**Supplementary table 3. Transcript levels of fibrogenic and HIF-α target genes in mice**

| Target gene | BDL | | | | CCl_4_ | | | |
| --- | --- | --- | --- | --- | --- | --- | --- | --- |
|  | Ad-Null | Ad-VHL+  Ad-HIF-1α TM | Ad-VHL+  Ad-HIF-2α TM | Ad-VHL | Ad-Null | Ad-VHL+  Ad-HIF-1α TM | Ad-VHL+  Ad-HIF-2α TM | Ad-VHL |
| Collagen-1α | 1.00±0.19 | 0.63±0.15* | 0.70±0.13* | 0.27±0.07*#⦁ | 1.00±0.19 | 0.55±0.15* | 0.62±0.18* | 0.26±0.06*#⦁ |
| TGF-β1 | 1.00±0.18 | 0.93±0.20 | 0.63±0.11*# | 0.49±0.13*# | 1.00±0.21 | 0.86±0.15 | 0.59±0.13*# | 0.37±0.09*#⦁ |
| TIMP-1 | 1.00±0.15 | 1.41±0.30* | 0.40±0.08*# | 0.26±0.07*# | 1.00±0.24 | 0.80±0.24 | 0.48±0.06*# | 0.32±0.10*# |
| PAI-1 | 1.00±0.16 | 0.93±0.19 | 0.51±0.11*# | 0.41±0.08*# | 1.00±0.27 | 1.89±0.39* | 0.59±0.21*# | 0.43±0.11*# |
| PDGF-B | 1.00±0.26 | 0.39±0.07* | 0.69±0.18*# | 0.34±0.06*⦁ | 1.00±0.15 | 0.44±0.13* | 0.85±0.28# | 0.35±0.07*⦁ |
| Glut1 | 1.00±0.20 | 1.68±0.23* | 0.34±0.10*# | 0.29±0.06*# | 1.00±0.24 | 2.36±0.54* | 0.12±0.03*# | 0.11±0.03*# |
| LDHA | 1.00±0.15 | 4.03±0.73* | 1.25±0.21# | 0.80±0.17# | 1.00±0.21 | 2.17±0.55* | 0.33±0.07*# | 0.24±0.05*# |
| PDK1 | 1.00±0.21 | 0.68±0.17* | 0.36±0.05*# | 0.17±0.03*# | 1.00±0.22 | 1.28±0.25 | 0.53±0.15*# | 0.42±0.09*# |
| EPO | 1.00±0.18 | 0.60±0.14* | 3.27±0.60*# | 0.50±0.11*⦁ | 1.00±0.24 | 0.12±0.03* | 5.97±1.26*# | 0.08±0.03*⦁ |

* p<0.01 vs Ad-Null, # p<0.01 vs Ad-VHL+ Ad-HIF-1α TM, ⦁ p<0.01Ad-VHL+Ad-HIF-2α TM.

**Figures**

**
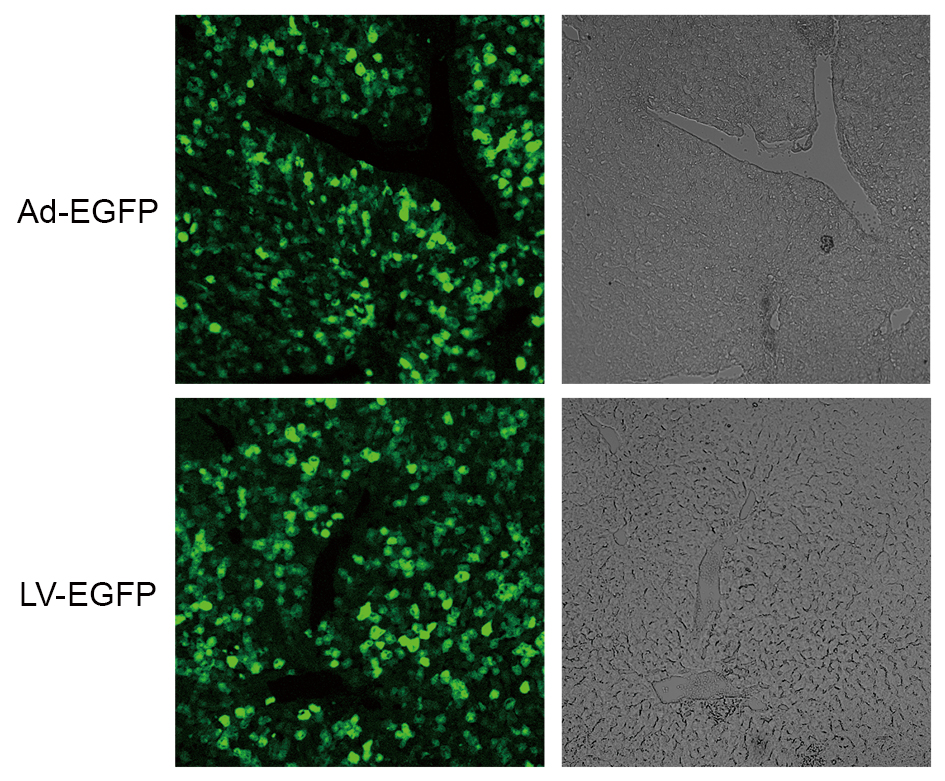
**

**Supplementary figure 1. Infection efficiencies of Ad-EGFP and LV-EGFP in vivo.** Representative frozen liver sections from mice injected with Ad-EGFP or LV-EGFP.

**A**


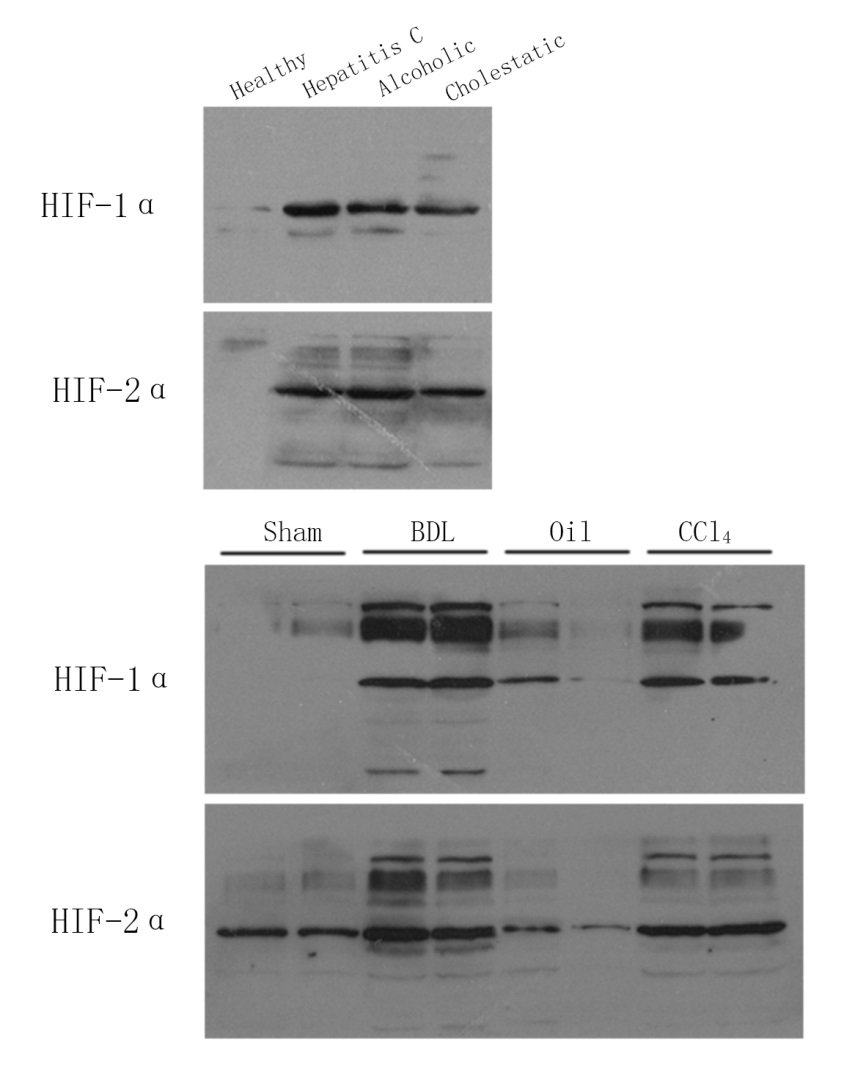


**B**

**Supplementary figure 2. Full scans of the blots of HIF-1α and HIF-2α.** (A) Western blot analysis of HIF-1α and HIF-2α expression in livers from fibrosis patients (hepatitis C, alcoholic, cholestatic). (B) Western blot analysis of HIF-1α and HIF-2α expression in livers from BDL- and CCl_4_-treated mice.


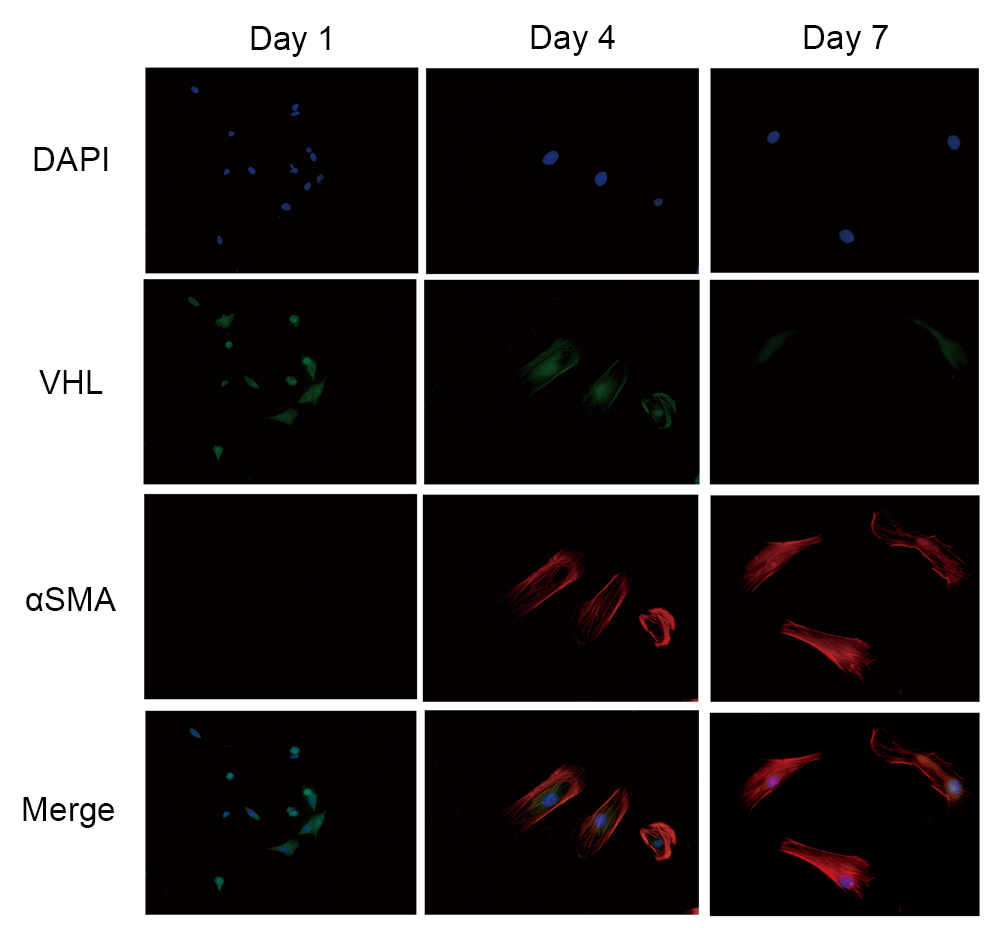


**Supplementary figure 3. VHL expression was decreased in self-activated HSCs in vitro.** Immunocytochemistry for VHL (green fluorescence) and αSMA (red fluorescence) in primary HSCs from mice after1, 4 or 7 days in culture.


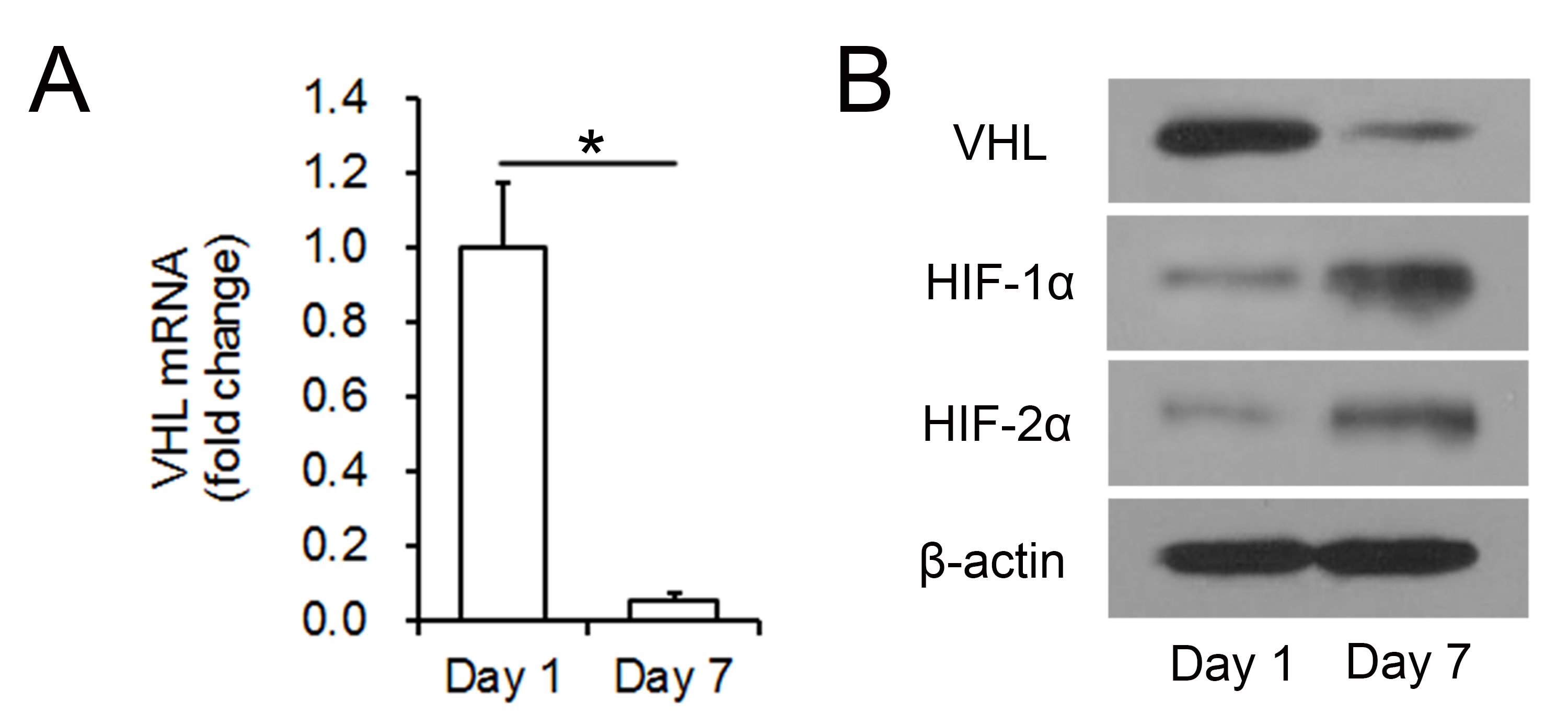


**Supplementary figure 4. VHL, HIF-1α and HIF-2α expression in self-activated HSCs in vitro.** (A) Real-time RT-PCR of VHL in primary HSCs from mice after1 or 7 days in culture (*p<0.001). (B) Western blot analysis of VHL, HIF-1α and HIF-2α expression in primary HSCs from mice after1 or 7 days in culture, and the gels have been run under the same experimental conditions.
